# How Have COVID-19 Isolation Policies Affected Young People’s Mental Health? – Evidence From Chinese College Students

**DOI:** 10.3389/fpsyg.2020.01529

**Published:** 2020-06-24

**Authors:** Bo Chen, Jinlu Sun, Yi Feng

**Affiliations:** ^1^Institute for Finance and Economics, Central University of Finance and Economics, Beijing, China; ^2^School of Humanities and Social Sciences, Beihang University, Beijing, China; ^3^Mental Health Center, Central University of Finance and Economics, Beijing, China; ^4^School of Psychology, Beijing Normal University Beijing, China

**Keywords:** COVID-19, isolation, young people, mental health, psychological interventions

## Abstract

The breakout of COVID-19 has brought about huge influence on people’s physic and mental health. This paper aims to investigate the mental health status of young people living in isolation due to the policy response to Coronavirus disease. Nine hundred ninety-two Chinese college students (*M*age = 19.45, *SD* = 1.41) were recruited to finish an online survey in the period of self-isolation. Seven dimensions of psychological well-being were measured, including mental status, knowledge of stress management, behavioral patterns, risk perception, academic stress, family relationships, and peer relationships. Results of cluster analysis indicated that young individuals’ mental status can be divided into three groups: high-risk (*n* = 61, *M*age = 19.26, *SD* = 1.32), medium-risk (*n* = 627, *M*age = 19.43, *SD* = 1.38), and low-risk (*n* = 304, *M*age = 19.54, *SD* = 1.49). Moreover, results of multiple regression showed that the isolation policy has had a complex influence on the symptoms of obsessive-compulsive disorder [*F*(12, 979) = 44.894, *p* < 0.001], fear [*F*(12, 979) = 30.776, *p* < 0.001], hypochondria [*F*(12, 979) = 22.530, *p* < 0.001], depression [*F*(12, 979) = 39.022, *p* < 0.001], and neurasthenia [*F*(12, 979) = 45.735, *p* < 0.001] via various factors. This paper also proposes a six-step intervention strategy to alleviate young people’s psychological problems while in isolation. It provides practical insights into the psychological interventions in face of the global threat.

## Introduction

The Coronavirus disease (COVID-19) outbreak that began in December 2019 has become a global threat. To control the rate of infection, several countries have adopted isolation strategies. The use of these strategies early in the development of infectious diseases has proven to be an effective prevention and control strategy (Yan and Zou, 2008), which can significantly reduce the number of susceptible and infected people (Zhou et al., 2019). The core logic of the isolation strategy is to reduce the spread of the pandemic by implementing social distancing in the local community (Glass et al., 2006). Faced with the spread of COVID-19, young people are an extremely vulnerable group. Research on pandemic influenza found that closing schools and imposing the requirement to stay at home reduces the infection rate by more than 90% (Glass and Glass, 2008). However, a long-term and strict isolation policy widely used to ensure social distancing will result in important changes to young individuals’ social networks and behaviors. For example, they may use mobile phones more to obtain information, resulting in internet addiction. This may cause young people to experience poor sleep quality (Liu et al., 2017) greater loneliness (Hidayati, 2019) and depressive symptoms (Jun, 2016) as well as a sense of estrangement from family, school, and peers, and even loss of self-control (Huang and Leung, 2009) or psychiatric disorders (Santos et al., 2015). Moreover, they also have to communicate with their families more often at close quarters, perhaps leading to family conflicts (Su et al., 2018). The social panic caused by COVID-19 is a growing catastrophe for young individuals, which may cause anxiety, affective disorders, post-traumatic stress disorder and a series of other adverse effects (Bolton et al., 2000). Through the application of school, family, community, and self-education programs, their ability to respond to the crisis could be effectively improved (Isen and Patrick, 1983; Codreanu et al., 2014). However, due to the isolation policy adopted by China and many countries to control the spread of the pandemic, it is not possible to provide timely crisis education and psychological intervention to young people through traditional school education and community programs. Thus, this paper proposes the following hypothesis: In the absence of effective school and community intervention, the COVID-19 isolation policy may affect the mental state of adolescents via factors including behavioral patterns, risk perception, knowledge of stress management, academic pressure as well as family and peer relationships. To analyze the impact of isolation policies and the spread of COVID-19 on adolescents’ mental health, this study conducted a questionnaire survey with 992 Chinese young people, analyzed their mental health situation, and proposed an effective intervention strategy.

## Materials and Methods

### Participants and Sampling

This study used a questionnaire survey to collect and analyze data from a campus in Henan Province, China. Random sampling was used to collect data via randomly distributed online questionnaire. Ever since Henan Province began the first-level response to the major public health emergency on January 25, 2020, all students have been required to isolate at home. This survey was conducted on March 27, 2020. The respondents to the questionnaire had been in isolation for more than 2 months. At the time of the investigation, Henan Province had reported a total of 1,272 confirmed cases of COVID-19 and 22 deaths.

### Ethical Approval and Consent

The participants were informed regarding the purpose and procedures of this survey via instructions at the head of the questionnaire. Informed written consent was provided on the first page of the questionnaire for all the participants. The research protocol was approved by the Ethics Committee of the Central University of Finance and Economics.

### Measures

The content of the questionnaire assessed seven dimensions of mental health: mental status, knowledge of stress management, behavioral patterns, risk perception, academic stress, family relationships as well as peer relationships.

#### Mental Status

Mental status was assessed according to five symptoms, namely, depression, neurasthenia, fear, obsessive-compulsive disorder (OCD), and hypochondria referring to the mental and behavioral questionnaire (Chen, 2002; Gao et al., 2004; World Health Organization [WHO], 2018). Participants were asked to rate their feelings during the outbreak of COVID-19 (e.g., less energy than before or no interest in anything) on a 5-point scale from 1 (not at all) to 5 (extremely). The cut-off scores were set 4 in this survey. Depression composite (α = 0.90) was assessed by six items, neurasthenia composite (α = 0.86) by five items, fear composite (α = 0.76) by six items, OCD composite (α = 0.86) by six items, and hypochondria composite (α = 0.75) by two items.

#### Behavioral Patterns

Behavioral patterns were divided into two types: positive response and negative response (Sato, 2005) and assessed by the *Brief Response Questionnaire* (Xie, 1999). Participants were asked to answer questions on a 5-point scale from 1 (strongly disagree) to 5 (strongly agree). The positive response composite (α = 0.79) consists of 10 items (e.g., You will relieve stress by working, studying or some other activities), and the negative response composite (α = 0.70) consists of seven items (e.g., You will relieve your worries by smoking, drinking, taking medicine, and eating).

#### Risk Perception

Due to the great uncertainty about the spread of the pandemic, risk perception of young individuals was divided into three types: anxiety, vulnerability, and controllability (Ajzen, 2002; Xie et al., 2005). Among them, anxiety refers to the degree of anxiety of pandemic, the degree of pandemic’s impact on individuals and society, and the continuity of pandemic’s consequences, representing the risk dimensions that have the greatest impact on the individual’s anxiety; Vulnerability refers to the estimates of the probability of respondents and the general population suffering from pandemic; Controllability refers to the sense of control of pandemic and the degree of mastery of pandemic related knowledge.

Similarly, participants were asked to answer the questions on a 5-point scale from 1 to 5. The composite of anxiety (α = 0.73) with six items (e.g., The COVID-19 epidemic is very worrying to me), controllability (α = 0.63) with three items (e.g., The COVID-19 epidemic is uncontrollable for the entire society) and vulnerability (α = 0.59) with three items (e.g., The general public is very likely to be infected with COVID-19) were separately computed.

#### Knowledge of Stress Management (KSM)

In addition, the questionnaire introduced factors such as knowledge of stress management. Good knowledge of stress management may help alleviate mental health problems. It was assessed by three items (e.g., How much do you know about the adverse reactions caused by stress? How much do you know about stress relief? How much do you know about pressure conduction?) with a 5-point scale from 1 to 5, with higher scores indicating more knowledge of stress management. The composite of KSM was computed with high reliability (α = 0.85).

#### Academic Pressure, Family, and Peer Relationships

The study also measured academic pressure (Findley and Cooper, 1983) family relationships and peer relationships. Academic pressure caused by the delay in starting school due to the isolation policy may aggravate their psychological problems. We measured the young’s academic pressure by the question that “How do you think the impact of pandemic (such as delayed start) on your studies?” with a 5-piont scale. Family and peer relationships can also affect young individuals’ psychological state. Control variables included gender, age, and the desire for intervention. We measured the two variables by the question that “How do you think the impact of pandemic on your family relationship (including relationship with parents, siblings)?” and “How do you think the impact of pandemic on your classmate relationship?”

### Data Analysis

First, we examined the normality of continuous variables with the Shapiro-Wilk test, and found that all continuous variables were non-normally distributes, so Spearman correlations were conducted. Second, a cluster analysis was performed based on mental status scores. Third, five multiple regression models were constructed, which included explained variables related to depression, neurasthenia, fear, OCD and hypochondria. The explanatory variables included positive response, negative response, anxiety, controllability, vulnerability, knowledge of stress management, academic pressure, family relationships, and peer relationships in addition to the control variables mentioned above. According to the regression model results, the study proposes a psychological intervention program. We set the significance level as 0.05 in this study.

## Results

### Demographic Characteristics and Correlations

A total of 992 questionnaires were randomly distributed, and all samples were valid; thus, the response rate was 100%. The final sample comprised 992 college students: Mean age = 19.45 ± 1.41 years; 468 (47.2%) were male (Mage = 19.28 ± 1.03 years), 524 (52.8%) were female (Mage = 19.61 ± 1.66 years). Significant correlations were found between the explanatory variable and the mental status (see Table 1).

**TABLE 1:** Descriptive statistics and spearman correlations.

	*M*	*SD*	1	2	3	4	5	6	7	8	9	10	11	12	13	14	15	16	17
1. Gender	1.53	0.50	1																
2. Age	19.45	1.41	0.02	1															
3. Intervention desire	2.00	1.02	0.02	0.02	1														
4. Positive response	3.67	0.51	0.08**	0.06	0.09**	1													
5. Negative response	2.71	0.61	−0.07*	–0.04	0.10**	–0.05	1												
6. Anxiety	2.98	0.68	0.02	0.05	0.20**	0.02	0.20**	1											
7. Controllability	1.88	0.71	–0.03	0.07*	0.17**	−0.21**	0.21**	0.31**	1										
8. Vulnerability	1.65	0.58	–0.05	0.04	0.22**	−0.13**	0.17**	0.30**	0.51**	1									
9. KSM	2.98	0.82	–0.03	0.03	0.15**	0.33**	−0.09**	−0.09**	−0.14**	–0.03	1								
10. Academic pressure	3.34	0.93	−0.18**	−0.08*	0.14**	−0.08*	0.14**	0.28**	0.14**	0.09**	−0.10**	1							
11. Family relationship	2.43	1.17	0.05	–0.01	0.25**	0.00	0.09**	0.22**	0.16**	0.19**	0.04	0.22**	1						
12. Peer relationship	2.34	1.07	0.00	0.02	0.26**	–0.06	0.07*	0.26**	0.18**	0.20**	0.04	0.24**	0.54**	1					
13. Depression	2.40	0.87	−0.12**	–0.05	0.14**	−0.25**	0.37**	0.28**	0.30**	0.24**	−0.17**	0.31**	0.25**	0.25**	1				
14. Neurasthenia	2.21	0.89	−0.10**	–0.04	0.17**	−0.32**	0.38**	0.25**	0.35**	0.31**	−0.18**	0.26**	0.22**	0.24**	0.77**	1			
15. Fear	2.73	0.74	0.02	0.02	0.29**	–0.00	0.24**	0.45**	0.24**	0.29**	–0.05	0.18**	0.22**	0.22**	0.34**	0.39**	1		
16. OCD	1.53	0.61	–0.03	0.00	0.34**	−0.19**	0.28**	0.31**	0.39**	0.40**	–0.01	0.16**	0.25**	0.26**	0.44**	0.53**	0.55**	1	
17. Hypochondria	1.40	0.72	–0.06	0.00	0.23**	−0.10**	0.15**	0.24**	0.29**	0.37**	0.02	0.09**	0.11**	0.12**	0.21**	0.26**	0.36**	0.53**	1

*p < 0.05, **p < 0.01.

### Cluster Analysis

**FIGURE 1:**
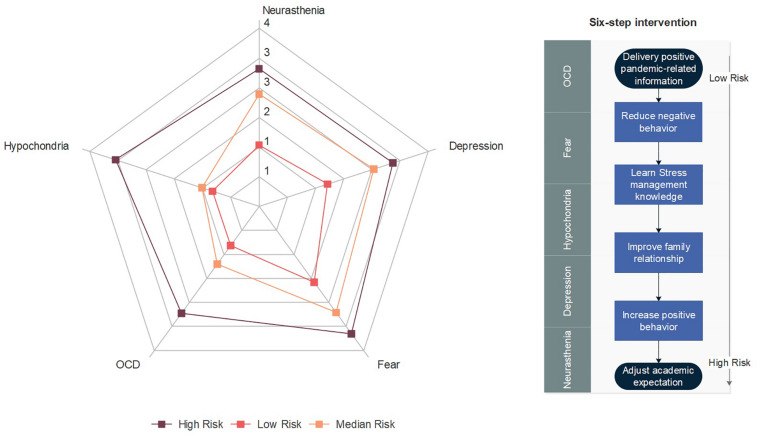
Cluster analysis results and the six-step intervention strategy.

Hierarchical cluster analysis of the mental status of 992 respondents found that the young people of Henan Province could be divided into three groups: 61 (6%) were high-risk individuals, 627 (63%) were medium-risk and 304 (31%) were low-risk individuals. The most important high-risk mental health symptoms were fear (*Mean* = 3.54 ± 0.62), hypochondria (*Mean* = 3.39 ± 0.87), depression (*Mean* = 3.17 ± 0.70) and neurasthenia (*Mean* = 3.09 ± 0.82); the primary middle-risk group symptoms were reflected in fear (*Mean* = 2.95 ± 0.59), depression (*Mean* = 2.71 ± 0.74) and neurasthenia (*Mean* = 2.52 ± 0.77); the main low-risk group symptoms were reflected in fear (*Mean* = 2.11 ± 0.63) and depression (*Mean* = 1.62 ± 0.55). The results of ANOVA showed that the three groups significantly differentiated in all the five symptoms: depression [*F*(2, 989) = 298.08, *p* < 0.001], neurasthenia [*F*(2, 989) = 337.78, *p* < 0.001], fear [*F*(2, 989) = 252.95, *p* < 0.001], OCD [*F*(2, 989) = 504.13, *p* < 0.001], hypochondria [*F*(2, 989) = 558.82, *p* < 0.001]. Figure 1 presented a radar chart of the mental status of these three types of young people.

### Multiple Regression Analysis

The study conducted a regression analysis of five mental health symptoms (Table 2). We found that all the five models were significantly predicted by the independent variables (*p* < 0.001). The adjusted R-square of the five models was from 0.207 to 0.374, demonstrating good explanatory power. According to the regression results, negative response affected all five mental health symptoms significantly; for example, bad behavior during isolation such as drinking and smoking leads to more serious psychological problems. Positive behavior can effectively relieve three of these symptoms including OCD (*B* = −0.157, *p* < 0.001), neurasthenia (*B* = −0.398, *p* < 0.001), and depression (*B* = −0.287, *p* < 0.001).

**TABLE 2:** Multiple regressions for the five mental health symptoms.

Models	Fear				Hypochondria				OCD				Neurasthenia				Depression			
Variables	*B*	*Beta*	*t*	*p*	*B*	*Beta*	*t*	*p*	*B*	*Beta*	*t*	*p*	*B*	*Beta*	*t*	*p*	*B*	*Beta*	*t*	*p*
(Constant)	0.413		1.162	0.245	0.158		0.446	0.656	0.284		1.032	0.302	1.821		4.577	**0.000**	2.011		5.029	**0.000**
Gender	0.042	0.028	1.006	0.315	–0.073	–0.051	–1.741	0.082	–0.006	–0.005	–0.184	0.854	–0.081	–0.045	–1.706	0.088	–0.109	–0.062	–2.292	**0.022**
Age	0.005	0.009	0.318	0.751	–0.003	–0.007	–0.230	0.818	0.000	0.000	0.007	0.994	–0.014	–0.021	–.826	0.409	–0.033	–0.053	–1.991	**0.047**
Tendency to intervene	0.123	0.167	5.603	**0.000**	0.088	0.125	4.037	**0.000**	0.112	0.186	6.615	**0.000**	0.043	0.049	1.759	0.079	0.008	0.010	0.335	0.737
Academic competition	0.040	0.050	1.650	0.099	0.005	0.007	0.214	0.830	0.016	0.024	0.844	0.399	0.118	0.123	4.317	**0.000**	0.129	0.137	4.705	**0.000**
Family relationship	0.036	0.057	1.721	0.086	0.016	0.026	0.768	0.443	0.037	0.070	2.261	**0.024**	0.080	0.105	3.390	**0.001**	0.083	0.112	3.511	**0.000**
Peer relationship	0.017	0.025	0.732	0.465	–0.023	–0.034	–0.968	0.333	0.011	0.019	0.586	0.558	0.040	0.048	1.521	0.129	0.052	0.064	1.973	**0.049**
KSM	–0.049	–0.054	–1.799	0.072	0.052	0.059	1.900	0.058	0.044	0.059	2.089	**0.037**	–0.091	–0.084	–2.987	**0.003**	–0.086	–0.082	–2.807	**0.005**
Positive response	0.052	0.036	1.219	0.223	–0.058	–0.041	–1.347	0.178	–0.157	–0.132	–4.705	**0.000**	–0.398	–0.231	–8.275	**0.000**	–0.287	–0.170	–5.928	**0.000**
Negative response	0.147	0.120	4.205	**0.000**	0.086	0.073	2.471	**0.014**	0.182	0.181	6.708	**0.000**	0.409	0.280	10.428	**0.000**	0.386	0.270	9.786	**0.000**
Anxiety	0.328	0.298	9.292	**0.000**	0.110	0.104	3.118	**0.002**	0.042	0.047	1.550	0.121	0.033	0.025	0.824	0.410	0.117	0.091	2.933	**0.003**
Controllability	0.015	0.014	0.420	0.674	0.160	0.158	4.459	**0.000**	0.199	0.230	7.154	**0.000**	0.149	0.118	3.696	**0.000**	0.104	0.084	2.568	**0.010**
Vulnerability	0.121	0.095	2.870	**0.004**	0.262	0.214	6.211	**0.000**	0.187	0.178	5.688	**0.000**	0.153	0.101	3.237	**0.001**	0.086	0.058	1.802	0.072
***F***			30.776	0.000			22.530	0.000			44.894	0.000			45.735	0.000			39.022	0.000
***N***			992				992				992				992				992	
***R*^2^**			0.274				0.216				0.355				0.359				0.324	
Adjusted *R*^2^			0.265				0.207				0.374				0.351				0.315	

*The gender was coded as “1 = male,” “2 = female.” The bold values indicate the significant p values at 0.05 level.*

The impact of anxiety, controllability and vulnerability indicated that when young people receive excessively negative pandemic information, it leads to more grave psychological problems. Therefore, when sending outbreak information to young people, more gentle and positive strategies should be adopted to avoid excessively negative communication. It is worth mentioning that family relationships can have an adverse effect on the three symptoms of OCD (*B* = 0.037, *p* < 0.05), neurasthenia (*B* = 0.080, *p* < 0.001), and depression (*B* = 0.083, *p* < 0.001). The staying at home policy causes young people to spend more time with their parents and makes these relationships more susceptible to conflict, which exacerbates the above three symptoms.

Conversely, developing the knowledge level of stress management has improved the two symptoms of neurasthenia (*B* = −0.091, *p* < 0.01), and depression (*B* = −0.086, *p* < 0.01). But it is not effective for fear and hypochondria, and even shows the opposite effect for OCD (*B* = 0.044, *p* < 0.05). This is because OCD is closely related to mental stress, and patients usually have a good understanding of stress management, but according to existing research, it is still difficult to alleviate the symptoms of OCD by simply improving the cognitive level of stress (Enright, 1991; Rufer et al., 2006). Meanwhile, the negative impact of academic stress on depression (*B* = −0.118, *p* < 0.001) and neurasthenia (*B* = −0.129, *p* < 0.001) was extremely significant but had no significant effect on other symptoms. In addition, gender (*B* = −0.109, *p* < 0.05) and age (*B* = −0.033, *p* < 0.05) only affected depression, with male and younger students more prone to depression.

## Discussion

This study found that the long-term isolation policy in response to COVID-19 has had a complex influence on the mental health of young people, which is consistent with Bolton et al. (2000) and Gao et al. (2004). This study also proved that it is difficult to really change the behavior patterns of adolescents only through strengthening knowledge education (Codreanu et al., 2014) and may even have negative effects on OCD symptoms. Moreover, the regression results also showed that the young people have expressed a strong desire to intervene, which further illustrated that there is a large room for improvement in the existing crisis management education. Hence, this paper proposes a six-step intervention strategy to alleviate the psychological problems of young individuals based on the influence of each factor on the five mental health symptoms (Figure 1). The first step proposed is the delivery of positive epidemic-related information to optimize the risk perception of young people by appealing to the two dimensions of anxiety and controllability. The second step is to improve their symptoms by reducing the opportunities for negative behavior. The third step is to improve their knowledge of stress management, while the fourth step is to alleviate family conflicts and improve family relationships. The fifth step involves cultivating positive behavioral habits, and the sixth step includes adjusting academic expectations. In practice, it is not necessary to implement all these measures for all young people, but a step-by-step intervention method should be adopted. For large-scale interventions, the first three steps could be delivered through online courses; however, for individuals with more serious psychological problems, the latter three measures should be further adopted.

The research has the following limitations. First, the survey was conducted in Henan, China. Due to cultural differences, different patterns may exist in other areas, so cross-cultural comparative studies are needed. Second, this survey was completed in March and there is no baseline for comparison. Hence, longitudinal studies can be carried out to provide a better picture of the impact on mental health of young people. Third, this paper only proposed a framework for intervention, and the specific intervention measures can be further explored in the future.

## Data Availability Statement

The datasets for this article are not publicly available because the data is only authorized for this study. Requests to access the datasets should be directed to JS, jinlu@buaa.edu.cn.

## Ethics Statement

The studies involving human participants were reviewed and approved by the Ethics Committee of the Central University of Finance and Economics. Written informed consent to participate in this study was provided by the participants’ legal guardian/next of kin.

## Author Contributions

BC: research design and manuscript writing. JS: data collection and modeling. YF: data processing. All authors contributed to the article and approved the submitted version.

## Conflict of Interest

The authors declare that the research was conducted in the absence of any commercial or financial relationships that could be construed as a potential conflict of interest.
